# Monitoring of Volatile Compounds of Ready-to-Eat Kiwifruit Using GC-IMS

**DOI:** 10.3390/foods12244394

**Published:** 2023-12-06

**Authors:** Jiajia Yuan, Hongbo Li, Shangqiao Cao, Zhenbin Liu, Na Li, Dan Xu, Haizhen Mo, Liangbin Hu

**Affiliations:** 1 School of Food and Biological Engineering, Shaanxi University of Science and Technology, Xi’an 710021, China; yuanjiajia@sust.edu.cn (J.Y.); shangqiao@sust.edu.cn (S.C.); zhenbinliu@sust.edu.cn (Z.L.); xudan@sust.edu.cn (D.X.); mohz@sust.edu.cn (H.M.); hulb@sust.edu.cn (L.H.); 2 College of Food Science and Engineering, Central South University of Forestry and Technology, Changsha 410004, China; linaznl@163.com

**Keywords:** ready-to-eat kiwifruit, GC-IMS, volatile organic compounds (VOCs), quality indexes

## Abstract

Ready-to-eat kiwifruit has gained significant market value in recent years due to its convenience and the increasing consumer demand for healthy ready-to-eat snacks. The volatile compound content (VOC) in ready-to-eat kiwifruit is a crucial factor determining its flavor and aroma. VOC is an important characteristic that positively affects the overall evaluation of ready-to-eat kiwifruit. In this study, we utilized gas chromatography-ion mobility spectrometry (GC-IMS) to investigate changes in the composition of VOCs in ready-to-eat kiwifruit during different storage periods (every 12 h). Our results revealed the presence of 55 VOCs in ready-to-eat kiwifruit, with alcohols, esters, and ketones being the dominant compounds responsible for the aromatic flavor. Among these compounds, methyl caproate, ethyl butyrate, and ethyl propionate provided specific fruit flavors to ready-to-eat kiwifruit, whereas esters played a secondary role. Furthermore, varying trends were observed for different compound types as the storage period increased: alcohols exhibited a decreasing trend, whereas ester products and some sulfur-containing compounds showed an increase. Additionally, fingerprint profiles of volatile compounds were established for each storage period, enabling the identification of characteristic substances. This comprehensive analysis of volatile flavor substances during the ripening of ready-to-eat kiwifruit will greatly contribute to enhancing its sensory quality, consumer appeal, and overall marketability.

## 1. Introduction

The concept of ‘ready-to-eat kiwifruit’ was developed to preserve kiwifruit after harvesting [1]. It refers to kiwifruit that has reached an edible state and is ready for immediate consumption upon purchase. The edibility and taste of kiwifruit are major concerns for consumers. One consistent drawback of domestic kiwifruit is the inability to consume them immediately [2]. Typically, purchased kiwifruits are hard, low in soluble solids, and sour in taste, making them unsuitable for direct consumption. They require natural ripening for a period of 7 to 10 days or more, or artificial ripening can be employed [3]. However, the longer they are stored, the greater the risk of water loss, wrinkling, rotting, and a decline in their eating quality. This greatly affects the consumer experience and discourages repeat purchases. Conversely, ready-to-eat kiwifruit eliminates the need for natural or artificial ripening and offers uniformly ripe flesh with superior flavor, enabling consumers to purchase and consume it immediately. The production of ready-to-eat kiwifruit is based on the rapid post-ripening process of kiwifruit by means of oxygenation and temperature control so that the fruit softens, the nutrient content is fully transformed, the sugar level is increased, and the flavor becomes optimal. Upon receipt, consumers can eat kiwifruit with uniform flesh ripeness and good flavor without having to wait. In addition, even for kiwifruit picked in the same batch, there are differences in the internal quality of the fruit (e.g., volatile compound composition). In addition to being bought and consumed immediately, ready-to-eat kiwifruit must have a certain “shelf life”, meaning that it remains edible for 5–7 days, which puts higher demands on the intrinsic quality and integrity of the ready-to-eat kiwifruit.

During fruit storage, changes in fruit quality and consumption are influenced by changes in the volatile organic matter content (VOC) of the fruit. Currently, VOCs are chosen as important indicators for investigating changes in fruit quality during storage. A study analyzed the characteristics of the new apple variety “Meihong” after picking and storage and found that, during storage at room temperature, the content of alcohols, esters, and ethylene gradually increased, and that the increase in the content of ethylene reduced the quality of apples, and apples also appeared to be softened; a series of results concluded that apples should be consumed within 10 days at room temperature [4]. Similar to apples, kiwifruit ripening relies on a variety of volatile organic compounds (VOCs), which are the main source of flavor and aroma in ready-to-eat kiwifruit [5]. These VOCs can be influenced by various factors, including storage time, temperature, and more [6]. Therefore, comprehending their composition and changes is essential for ensuring the quality and taste of ready-to-eat kiwifruit. Numerous studies have demonstrated that kiwifruit contains a diverse range of VOCs such as alcohols, esters, monoterpenes, and ketones [7]. Notably, a significant proportion of these VOCs consist of alcohols including ethanol, isoamyl alcohol, methanol, and isobutanol, which contribute to the strong aromatic flavor of kiwifruit. Esters play a secondary role, with compounds like hexyl acetate, ethyl butyrate, and ethyl valerate providing specific fruit flavors to kiwifruit [8].

Gas chromatography-ion mobility spectrometry (GC-IMS) is a technique that combines two methods, gas chromatography and ion mobility spectrometry, enabling rapid analysis of volatile compounds [9,10,11]. GC-IMS is employed for the detection and quantification of volatile organic compounds in air, liquids, or solids. In the field of food analysis, it has become widely utilized for various purposes, including flavor and quality analysis, trace toxic chemical detection, and identification of adulteration. This technique offers several advantages, such as high sensitivity, non-destructive analysis, rapid results, high reliability, ease of use, and cost-effectiveness [12]. For instance, GC-IMS has been employed in flavor analysis to assess VOC production in different fruits such as pomegranate [13], passion fruit [14], and peach [15]. Additionally, it has been used for comparative analysis to identify unknown flavors in various meats, including donkey meat and shrimp (crayfish) meat [16,17].

The utilization of GC-IMS for analyzing the fluctuation of volatile compounds in kiwifruit holds significant importance in elucidating the flavor and aroma aspects of ready-to-eat kiwifruit during various storage periods. Although some researchers have conducted comparative analyses on the aroma characteristics of different kiwifruit varieties [18], studies are limited. Therefore, in this study, GC-IMS was used to assess the changes in the content of different volatile organic compounds in ready-to-eat kiwifruit and to establish the fingerprints of volatile compounds at each storage period to identify the characteristic substances. Finally, PCA and correlation analysis were used to establish the relationship between the content of volatile organic compounds and quality in ready-to-eat kiwifruit, and to screen out the ready-to-eat kiwifruit with the highest flavor profile within a certain storage period.

## 2. Materials and Methods

### 2.1. Materials

‘XuXiang’ ready-to-eat kiwifruits used in the experiment came from qifeng fruit industry Co., Ltd. (Meizhou, Meixian, Shaanxi Province, China). Ready-to-eat kiwifruits were selected on the basis of uniform size and shape. Further, sound fruits were selected without damages and blemishes. Kiwifruits were divided into 8 groups and each group comprised 3 fruits. The samples of kiwifruits were stored at −20 °C.

### 2.2. Preparation of Samples

A set of ready-to-eat kiwifruit samples (3 kiwifruit per sample) was selected at a time, then peeled and homogenized to obtain the pulp. The ready-to-eat kiwifruit pulp was rapidly frozen in liquid nitrogen, and the frozen ready-to-eat kiwifruit was crushed using a grinder. Two grams of ready-to-eat kiwifruit powder and 0.5 g of saturated sodium chloride solution were taken into sample bottles for analysis.

### 2.3. GC-IMS Analysis

The volatile components of ready-to-eat kiwifruit samples were analyzed using gas chromatography combined with ion mobility spectrometry (Flavour Spec GC-IMS) (G.A.S, Essen, Germany). Approximately 0.1 mL of sample from each ready-to-eat kiwifruit was placed into a headspace bottle and incubated at 40 °C for 20 min at 500 rpm before determination. The injection temperature was 85 °C. In non-diverting mode, the cleaning time is 30 s.

The GC detection conditions were as follows [19]: MXT-5 column (15 m × 0.53 mm × 1 μm); column temperature 60 °C, carrier gas is high-purity nitrogen (≥99.999%); 0–2 min, E1 flow rate 150 mL/min, E2 flow rate 2 mL/min; 2–10 min, E1 flow rate 150 mL/min, E2 flow rate linearly increased to 10 mL/min; 10–20 min, E1 flow rate 150 mL/min, E2 flow rate linearly increased to 100 mL/min; for 20–30 min, E1 flow velocity was 150 mL/min, and E2 flow velocity was linearly increased to 150 mL/min. The IMS detection conditions were as follows: length of drift tube is 5 cm, drift tube temperature is 45 °C, drift gas is high purity nitrogen (≥99.999%), flow rate is 150 mL/min, and detector temperature is 45 °C.

### 2.4. Data Analysis

The reference of data analysis is given here. The qualitative and quantitative analysis of the analytical spectrum and data can be viewed below. The NIST database and IMS database built-in software was used for a qualitative analysis of substances [20]. Each point in the figure represents a volatile component, and quantitative analysis was performed after the establishment of a standard curve. The Reporter plug-in was used to directly compare the spectral differences between the samples (3D spectrum, 2D top view and differential spectrum). The Gallery Plot plug-in was used for fingerprint comparison to compare volatile component differences visually and quantitatively between different samples.

## 3. Results

### 3.1. GC-IMS Topography of Ready-to-Eat Kiwifruit Samples at Different Storage Periods

**Figure 1:**
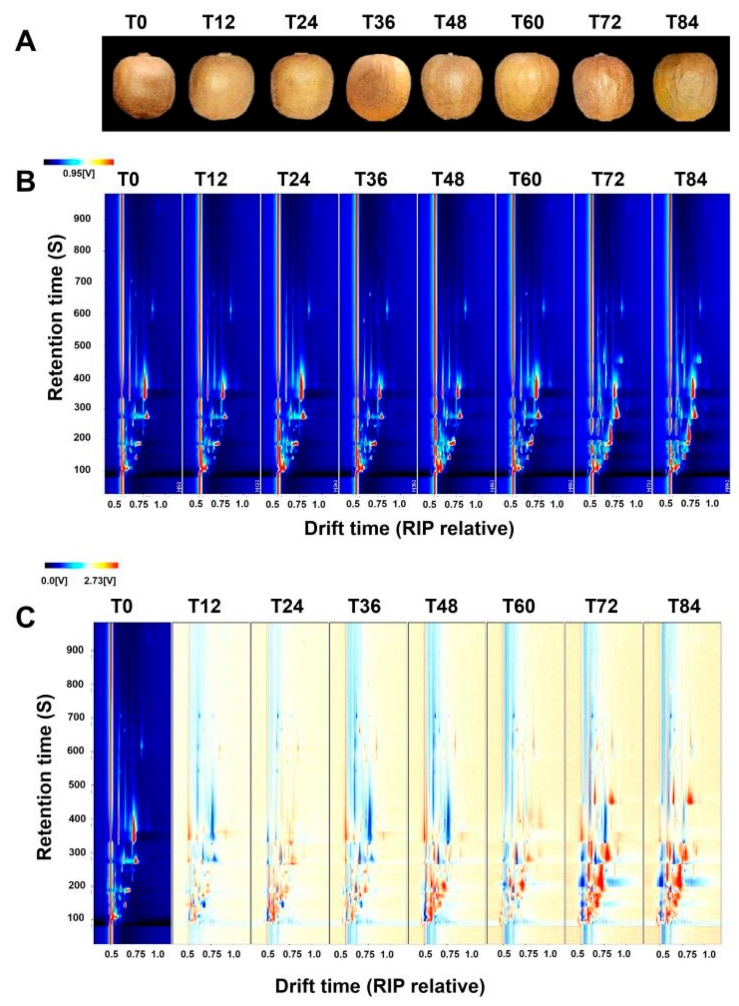
Ready-to-eat kiwifruit samples and GC-IMS topography of ready-to-eat kiwifruit samples at different storage periods. (**A**) Samples of ready-to-eat kiwifruit at different storage periods (tested every 12 h). (**B**) Top view of GC-IMS for VOCs in ready-to-eat kiwifruit (direct comparison). (**C**) GC-IMS for VOCs of ready-to-eat kiwifruit (comparison of differences).

During storage, ready-to-eat kiwifruit samples undergo various material changes, leading to the generation of various volatile organic compounds (VOCs) such as aldehydes, alcohols, ketones, and esters. The content of these VOCs may either increase or decrease during storage time. Samples of ready-to-eat kiwifruit at different storage periods are shown in Figure 1A. Figure 1A shows the peel variation of ready-to-eat kiwifruit at different storage periods (every 12 h). Within 84 h of storage, the ready-to-eat kiwifruit peel appeared slightly wrinkled at 48 h, and its wrinkling increased with time. This is because ready-to-eat kiwifruit contains a lot of water, but some of the water in the flesh is slowly lost during the process, making the kiwifruit skin wrinkled.

Pre-treatment of each group of samples was carried out via peeling, homogenization, freezing with liquid nitrogen, and milling to obtain ready-to-eat kiwifruit powder, which was analyzed by mixing with saturated sodium chloride in 10 mL sample bottles. VOCs present in ready-to-eat kiwifruit samples at different storage periods were analyzed using gas chromatography combined with a GC-IMS. Figure 1B illustrates the top view of the analysis results, depicted as a two-dimensional plot. Notably, several key features can be observed: (1) the entire figure background appears blue, while the red vertical line located at a horizontal coordinate of 1.0 represents the reactive ion peak (RIP) after normalization; (2) the vertical coordinate represents the retention time(s) of the gas chromatography, whereas the horizontal coordinate represents the ion migration time (normalized); (3) each point situated on both sides of the RIP peak represents a volatile component. The color of the point reflects the concentration of the substance, where white indicates lower concentration, red indicates higher concentration, and a darker color indicates a greater concentration. Evidently, most of the volatile compounds identified in ready-to-eat kiwifruit samples have retention times between 100 and 450 s and relative drift times ranging from 0.5 to 0.8 s. Figure 1C shows the variance analysis of the samples using GC-IMS, with RQHZ-2 serving as the standard against which other samples are compared. Difference maps were obtained via topographic derivation, using T0 as the background, to detect changes in VOCs within ready-to-eat kiwifruit during different storage durations. The signal gradually intensifies as the storage time of kiwis increases, particularly after T48, as observed by the presence of prominent red and blue signal retention time ranges from 100 to 500 s, as well as drift times of 0.5–0.9 s. Most signals shown in Figure 1C for the T48–T84 period significantly surpass those in the T0 group.

In summary, the results indicate that the VOCs in ready-to-eat kiwifruit undergo continuous changes during an extended storage time. These findings further suggest that the flavor of ready-to-eat kiwifruit is influenced by VOCs.

### 3.2. Identification of VOCs in Ready-to-Eat Kiwifruit Samples at Different Storage Periods

**Figure 2:**
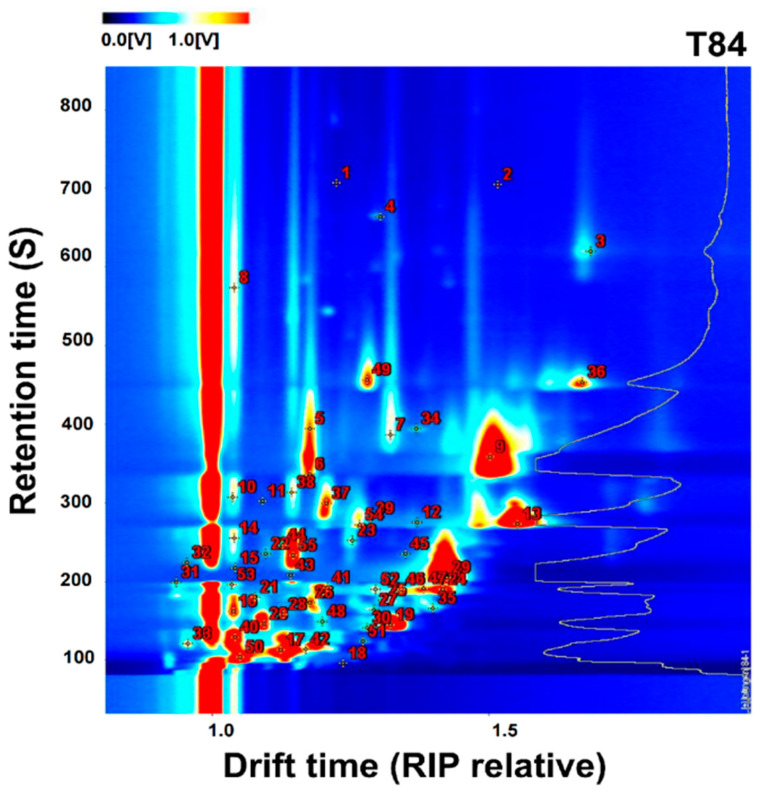
Qualitative results of gas-phase ion mobility spectra of ready-to-eat kiwifruit (T84).

The ready-to-eat kiwifruit samples underwent ionization in the column and were subsequently analyzed using the ion mobility rate system. The qualitative analysis of VOCs in ready-to-eat kiwifruit is shown in Figure 2, where the vertical coordinates indicate the retention time and the horizontal coordinates indicate the drift time. From the GC × IMS library, a total of 55 fractions were identified (Figure 2 and Table 1). These fractions encompassed various compounds, including seven alcohols, fourteen aldehydes, ten esters, seven ketones, three organic acids, two thiophenes, three furans, two alkenes, and three sulfur-containing compounds, dipropylene glycol monobutyl ether, pentamethylheptane, pyrrole, and 3-butenenitrile.

Firstly, alcohols and aldehydes are the most widely present and important compounds in ready-to-eat kiwifruit, and these compounds are usually the source of the grass flavor produced by ready-to-eat kiwifruit [21]. Among them, (E)-2-hexen-1-ol-D has the strongest signal, i.e., the highest content, as well as Hexanal-D, 2-Hexanol, etc., which may be responsible for the relatively strong grass flavor of ready-to-eat kiwifruit. Secondly, esters are volatile compounds that produce fruity and sweet aromas in kiwifruit [22]. There were five straight-chain esters in the ready-to-eat kiwifruit samples, namely Ethyl acetate, Ethyl butyrate, Ethyl propanoate, Hexanoic acid methyl ester, and Butanoic acid methyl ester, with Ethyl acetate being more abundant. Moreover, some ketones, acids, and other compounds, which change in small amounts during the ripening of ready-to-eat kiwifruit, such as 5-Ethyldihydro-2(3H)-furanone and gamma-Terpinene, can be shown to make a small contribution to the aroma of ready-to-eat kiwifruit.

However, during the passage through the drift region, certain individual compounds generated multiple signals due to the occurrence of adduct formation between the analyzed ions and neutral molecules (such as dimers and trimers). For instance, (E)-2-Hexen-1-ol, Hexanal, ethyl acetate, 1-penten-3-one, 2-pentenal (E), Pentanal, Butanoic acid methyl ester, and 2-ethylfuran exhibited two peaks each, attributable to the presence of both monomers and dimers (Table 1).

### 3.3. Fingerprint Study of VOCs in Ready-to-Eat Kiwifruit Samples at Different Storage Periods

**Figure 3:**
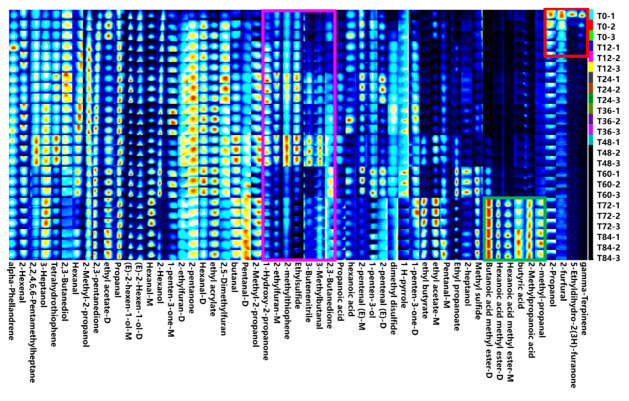
Fingerprint of ready-to-eat kiwifruit at different storage periods. The box lines in the graph represent all selected signal peaks in the samples. Each column in the graph represents the signal peaks of the same VOCs in different samples.

The fingerprint profile of ready-to-eat kiwifruit was established based on peak signals observed in the topography (Figure 3). In this profile, each row represents the signal peaks of a specific sample, while each column corresponds to the same substance across different samples. The content of VOCs is indicated by the shade of color in each cell, with darker shades representing higher concentrations. The red box within the profile signifies the characteristic region of ready-to-eat kiwifruit at T0, encompassing four volatile compounds: gamma-Terpinene, 5-Ethyldihydro-2(3H)-furanone, 2-furfural, and 2-Propanol. These compounds exhibited the highest abundance at T0, but their content decreased significantly with prolonged storage time. The green boxes depict the characteristic peaks of ready-to-eat kiwifruit at T72 and T84, which include 2-methyl-propanal, 2-methylpropanoic acid, butyric acid, Butanoic acid methyl ester-D, Hexanoic acid methyl ester-M, and Hexanoic acid methyl ester-D. These five volatile compounds were most abundant during the late storage period. Furthermore, the pink boxes illustrate the variation in content for seven characteristic volatile compounds: 2,3-Butanedione, 3-Methylbutanal, 3-Butenenitrile, Ethylsulfide, 2-methylthiophene, 2-ethylfuran-M, and 1-Hydroxy-2-propanone. These VOCs exhibited an initial rise in concentration at T0, followed by a peak at a certain storage period as time progressed. For instance, 1-Hydroxy-2-propanone peaked at T24 but gradually declined with increasing storage time.

In conclusion, the majority of VOCs in ready-to-eat kiwifruit exhibit an upward trend until reaching the stage of edibility. However, upon reaching the edible stage, certain VOCs demonstrate a decreasing trend (such as ketones), while others display an increasing trend (such as esters). These changes in VOCs serve as characteristic indicators of the storage period and can be utilized to determine the optimal consumption time and shelf life of ready-to-eat kiwifruit.

### 3.4. Principal Component Analysis and Correlation Study of VOCs in Ready-to-Eat Kiwifruit at Different Storage Periods

**Figure 4:**
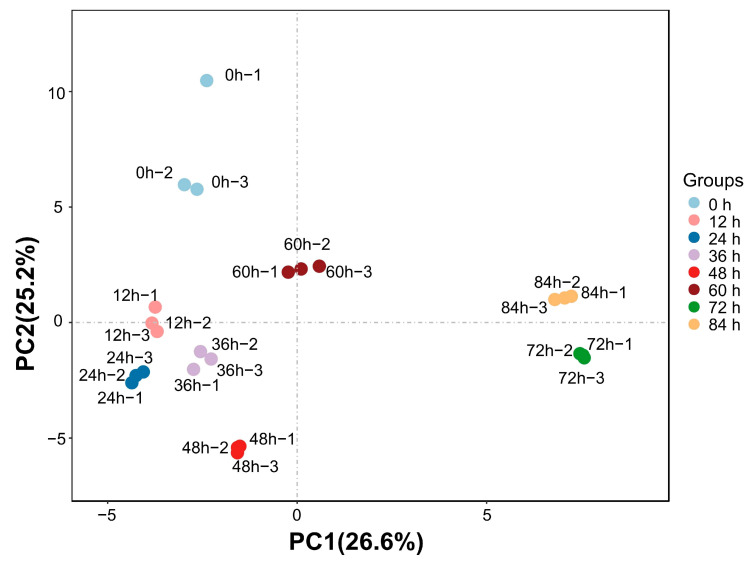
PCA analysis of VOCs in ready-to-eat kiwifruit samples.

PCA (Principal Component Analysis) is a basic technique for taking data to dimensionality reduction, usually applied in multivariate statistics [23]. Its primary objective is to replace multiple variables with a smaller set of variables while retaining most of the original information. PC1 and PC2 account for 36.6% and 25.2% of the data variance, respectively, contributing to a combined total of 61.8% variance. The PCA distribution map (Figure 4) reveals four distinct regions. Region I includes T12, T24, T36, and T48; Region II includes T0 and T60; Region III includes T84; and Region IV includes T72. However, there was a high degree of similarity between the ready-to-eat kiwifruit samples from Zones I and II in terms of the content of VOCs, and significant differences between Zones III and IV and the other zones. To summarize, changes in the quality of ready-to-eat ready-to-eat kiwifruit are manifested as changes in various volatile organic compounds (VOCs) and similar or significantly different changes over time. Combining Figure 3 and Figure 4, the significant difference in the content of volatile organic compounds in the ready-to-eat kiwifruit samples of Zones III and IV compared to the other zones is caused by the accumulation of the content of volatile organic compounds in the green boxes in Figure 3. It can be tentatively concluded that the quality of ready-to-eat kiwifruit changes significantly after 60 h, and, based on the changes, the optimal period of consumption can be selected.

**Figure 5:**
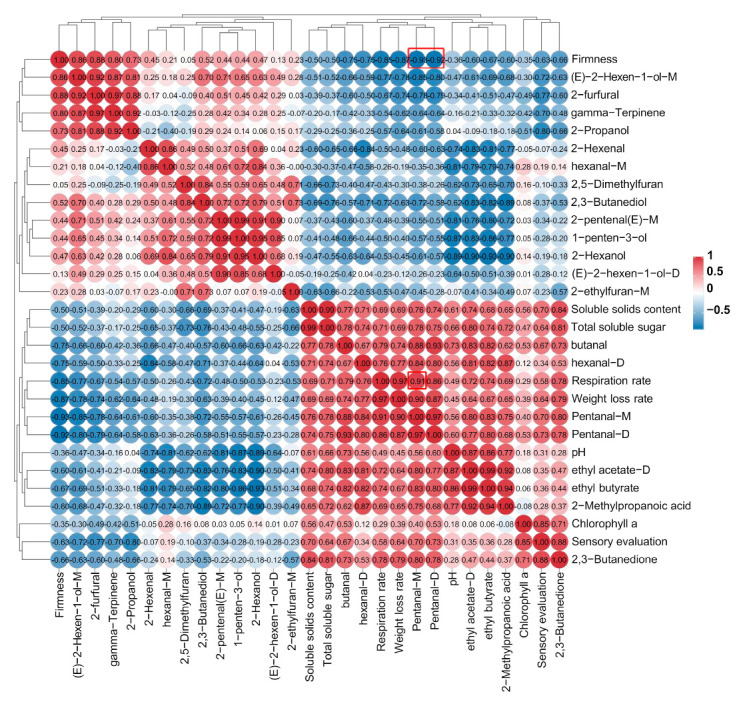
Correlation analysis of ready-to-eat kiwifruit quality indicators (firmness, soluble solids, total soluble sugars, pH, chlorophyll a, respiration rate, sensory evaluation, and weight loss rate) with VOCs.

In this study, we examined the correlation between the VOCs content of ready-to-eat kiwifruit and various quality indicators such as firmness, soluble solids content, total soluble sugar, pH, chlorophyll a, respiration rate, sensory evaluation, and weight loss rate (Figure 5). Positive correlations are represented by red, while negative correlations are indicated by blue. The intensity of the color reflects the strength of the correlation, with darker shades indicating higher levels of correlation. Notably, Pentanal-M exhibited a highly significant positive correlation with respiration rate (correlation coefficient: 0.91), as well as a significant negative correlation with firmness (correlation coefficient: −0.93). Similarly, Pentanal-D showed a significant negative correlation with hardness, as evidenced by a correlation coefficient of −0.92. As shown in Figure 5, most alcohol compounds (butanal, 3-Heptanol, and hexanal-D) displayed a significant positive correlation with the quality indicators. This indicates that aldehydes have a relatively high impact on the quality of ready-to-eat kiwifruit, and most of these aldehydes are formed by the oxidative degradation of ready-to-eat kiwifruit fatty acids. In addition, from the sensory evaluation, the accumulation of some volatile compounds affects the taste, such as gamma-Terpinene, (E)-2-hexen-1-ol, 2-Propanol, and 2-furfural, probably because too high a content produces a strong alcoholic flavor.

In summary, the correlation between the concentrations of different flavor compounds and quality indicators in ready-to-eat kiwifruit presents a novel approach for assessing the quality of ready-to-eat kiwifruit. By monitoring changes in volatile compound levels, it becomes possible to determine the optimal storage and edible conditions for ready-to-eat kiwifruit, thereby facilitating the rapid advancement of the ready-to-eat kiwifruit industry.

## 4. Discussion

Ready-to-eat kiwifruit, also known as pre-sliced or pre-packaged kiwifruit, can be made into a healthy snack either by packing them straight away or by peeling, slicing, and packaging them for immediate consumption. The market value of ready-to-eat kiwifruit is driven by various factors, including convenience [24], extended shelf life [25], and the increasing demand for healthy snacking options [26]. However, there are technical challenges involved in producing high-quality ready-to-eat kiwifruit while preserving its texture and nutritional value throughout processing and packaging stages [27]. It is crucial to maintain the fruit’s freshness and visual appeal without compromising taste and nutrient content. To achieve this, advanced processing techniques are necessary to minimize enzymatic browning (Yildiz et al., 2022), preserve firmness [28], and prevent the loss of essential nutrients, such as vitamin C [29].

To establish the relationship between volatile organic compounds and the ripening of ready-to-eat kiwifruit requires understanding the changes in volatile organic compound content during ripening of ready-to-eat kiwifruit [30]. These VOCs contribute to the characteristic aroma and flavor of ready-to-eat kiwifruit and influence the consumer acceptance of ready-to-eat kiwifruit. The content of VOCs undergoes dynamic changes during the ripening process of ready-to-eat kiwifruit. Several compounds, including esters, aldehydes, alcohols, and terpenes, contribute to the fruity, floral, and sweet flavors of the fruit [31]. Monitoring the levels of these VOCs at different ripening stages aids in determining the optimal time for harvesting and processing ready-to-eat kiwifruit. Consequently, this study aims to analyze the volatile flavor characteristics during ready-to-eat kiwifruit ripening to provide valuable insights for optimizing the ripening process of ready-to-eat kiwifruit. By closely observing the changes in volatile flavor compounds throughout ripening, researchers and producers can enhance their understanding of biochemical transformations occurring and develop effective strategies to attain the desired flavor and aroma profile in ready-to-eat kiwifruit.

## 5. Conclusions

In this study, the changes in the content of different volatile organic compounds (VOCs) in ready-to-eat kiwifruit were evaluated using the GC-IMS technique. The analysis of ready-to-eat kiwifruit samples revealed significant changes in the type and content of VOCs during storage; some compounds even existed in both monomeric and dimeric forms, e.g., hexanal, ethyl acetate, glutaraldehyde, and methyl butyrate. The analysis of the changes in the content of all VOCs revealed an increase in the content of VOCs such as aldehydes, ketones, and esters, with significant increases in Pentanal-D, 1-penten-3-one-D, ethyl acetate-M, Hexanoic acid methyl ester-M, and Hexanoic acid methyl ester-D, while the content of VOCs such as alcohols and hydrocarbons decreased, e.g., gamma-Terpinene and 2-Propanol. The PCA revealed that ready-to-eat kiwifruit reached the turning point for VOCs at T60, which is the turning point for ready-to-eat kiwifruit to reach the optimal consumption period, and that the characteristic substances 2-methyl-propanal, 2-methylpropanoic acid, butyric acid, Butanoic acid methyl ester-D, Hexanoic acid methyl ester-M, and Hexanoic acid methyl ester-D were found in the ready-to-eat kiwifruit. Correlation analysis revealed that the accumulation of esters (T72, T84), especially methyl butyrate-D, methyl caproate-M, and methyl caproate-D, promoted fruit aroma and sweetness, and the sensory evaluation of kiwifruit reached a high level. In conclusion, the optimal consumption period for ready-to-eat kiwifruit is between T72 and T84.

## Figures and Tables

**Table 1:** Information on the identified compounds in ready-to-eat kiwifruit samples.

Count	Compound	CAS#	Formula	MW	RI	Rt [sec]	Dt [a.u.]	Comment
1	gamma-Terpinene	C99854	C_10_H_16_	136.2	1061.5	706.697	1.22657	
2	5-Ethyldihydro-2(3H)-furanone	C695067	C_6_H_10_O_2_	114.1	1060.6	704.875	1.51882	
3	alpha-Phellandrene	C99832	C_10_H_16_	136.2	1014.2	619.226	1.6867	
4	hexanoic acid	C142621	C_6_H_12_O_2_	116.2	1039	663.569	1.30741	
5	(E)-2-Hexen-1-ol-M	C928950	C_6_H_12_O	100.2	891.6	394.266	1.17944	Monomer
6	2-Hexenal	C505577	C_6_H_10_O	98.1	848.9	335.965	1.1794	
7	3-Heptanol	C589822	C_7_H_16_O	116.2	885.7	385.69	1.32428	
8	2,2,4,6,6-Pentamethylheptane	C13475826	C_12_H_26_	170.3	989.1	572.835	1.04205	
9	(E)-2-hexen-1-ol-D	C928950	C_6_H_12_O	100.2	865.6	357.683	1.50484	Dimer
10	Tetrahydrothiophene	C110010	C_4_H_8_S	88.2	824.7	306.724	1.03885	
11	2-furfural	C98011	C_5_H_4_O_2_	96.1	820.2	301.595	1.09399	
12	2,3-Butanediol	C513859	C_4_H_10_O_2_	90.1	795.7	275.189	1.37285	
13	Hexanal-D	C66251	C_6_H_12_O	100.2	793.5	272.909	1.55403	Dimer
14	2-methylthiophene	C554143	C_5_H_6_S	98.2	776.2	255.052	1.042	
15	Ethylsulfide	C352932	C_4_H_10_S	90.2	736.1	216.68	1.04382	
16	1-Hydroxy-2-propanone	C116096	C_3_H_6_O_2_	74.1	655.6	161.513	1.04123	
17	2-Methyl-2-propanol	C75650	C_4_H_10_O	74.1	535.8	112.093	1.12703	
18	2-Propanol	C67630	C_3_H_8_O	60.1	482.8	95.364	1.23904	
19	ethyl acetate-D	C141786	C_4_H_8_O_2_	88.1	618.8	144.399	1.32483	Dimer
20	ethyl acetate-M	C141786	C_4_H_8_O_2_	88.1	620.2	144.976	1.09724	Monomer
21	1-penten-3-one-M	C1629589	C_5_H_8_O	84.1	690.4	179.974	1.08056	Monomer
22	2-pentenal (E)-M	C1576870	C_5_H_8_O	84.1	755.9	234.865	1.09932	Monomer
23	Hexanal-M	C66251	C_6_H_12_O	100.2	773.1	251.903	1.255	Monomer
24	ethyl acrylate	C140885	C_5_H_8_O_2_	100.1	702.9	189.337	1.41905	
25	1-penten-3-one-D	C1629589	C_5_H_8_O	84.1	682.6	175.372	1.31128	Dimer
26	Pentanal-M	C110623	C_5_H_10_O	86.1	676.8	172.299	1.17955	Monomer
27	Propanoic acid	C79094	C_3_H_6_O_2_	74.1	659.3	163.378	1.29486	
28	3-Butenenitrile	C109751	C_4_H_5_N	67.1	647	157.341	1.13159	
29	Butanoic acid methyl ester-D	C623427	C_5_H_10_O_2_	102.1	722.3	204.902	1.42727	Dimer
30	butanal	C123728	C_4_H_8_O	72.1	610	140.54	1.28211	
31	1-penten-3-ol	C616251	C_5_H_10_O	86.1	714.1	198.109	0.93668	
32	1 H-pyrrole	C109977	C_4_H_5_N	67.1	744.1	223.838	0.95648	
33	Methyl sulfide	C75183	C_2_H_6_S	62.1	558.4	120.078	0.95801	
34	2-heptanol	C543497	C_7_H_16_O	116.2	891.2	393.641	1.37094	
35	Pentanal-D	C110623	C_5_H_10_O	86.1	662.9	165.158	1.4016	Dimer
36	Hexanoic acid methyl ester-D	C106707	C_7_H_14_O_2_	130.2	927.8	452.939	1.67159	Dimer
37	ethyl butyrate	C105544	C_6_H_12_O_2_	116.2	817.2	298.219	1.20981	
38	butyric acid	C107926	C_4_H_8_O_2_	88.1	829.6	312.514	1.14656	
39	2-Hexanol	C626937	C_6_H_14_O	102.2	800	279.654	1.28805	
40	2-Methyl-2-propenal	C78853	C_4_H_6_O	70.1	580.5	128.447	1.04333	
41	2,3-pentanedione	C600146	C_5_H_8_O_2_	100.1	705.3	191.171	1.21055	
42	2,3-Butanedione	C431038	C_4_H_6_O_2_	86.1	539.2	113.262	1.17159	
43	Ethyl propanoate	C105373	C_5_H_10_O_2_	102.1	725.5	207.533	1.14509	
44	dimethyl disulfide	C624920	C_2_H_6_S_2_	94.2	767	245.679	1.12909	
45	2-pentenal (E)-D	C1576870	C_5_H_8_O	84.1	755.3	234.341	1.35143	Dimer
46	2,5-Dimethylfuran	C625865	C_6_H_8_O	96.1	702.7	189.174	1.34403	
47	2-pentanone	C107879	C_5_H_10_O	86.1	704.9	190.854	1.38444	
48	3-Methylbutanal	C590863	C_5_H_10_O	86.1	627.3	148.184	1.20127	
49	Hexanoic acid methyl ester-M	C106707	C_7_H_14_O_2_	130.2	929.6	456.029	1.28241	Monomer
50	Propanal	C123386	C_3_H_6_O	58.1	509	103.307	1.05229	
51	2-methyl-propanal	C78842	C_4_H_8_O	72.1	567.4	123.433	1.2756	
52	2-ethylfuran-D	C3208160	C_6_H_8_O	96.1	703.2	189.539	1.29837	Dimer
53	2-ethylfuran-M	C3208160	C_6_H_8_O	96.1	710.6	195.363	1.03755	Monomer
54	hexanal	C66251	C_6_H_12_O	100.2	791.8	271.209	1.26865	
55	2-Methylpropanoic acid	C79312	C_4_H_8_O_2_	88.1	753.9	232.987	1.14987	

Note: MW, molecular mass; RI, retention index; Rt, retention time; Dt, drift time.

## Data Availability

Data are contained within the article.
